# The Oesophageal Cancer Multidisciplinary Team: Can Machine Learning Assist Decision-Making?

**DOI:** 10.1007/s11605-022-05575-8

**Published:** 2023-01-23

**Authors:** Navamayooran Thavanesan, Ganesh Vigneswaran, Indu Bodala, Timothy J. Underwood

**Affiliations:** 1 School of Cancer Sciences, Faculty of Medicine, University of Southampton, University Hospitals Southampton, Southampton, UK; 2 School of Electronics and Computer Science, University of Southampton, Southampton, UK

**Keywords:** Machine learning, Artificial intelligence, Oesophageal cancer, Multidisciplinary team

## Abstract

**Background:**

The complexity of the upper gastrointestinal (UGI) multidisciplinary team (MDT) is continually growing, leading to rising clinician workload, time pressures, and demands. This increases heterogeneity or ‘noise’ within decision-making for patients with oesophageal cancer (OC) and may lead to inconsistent treatment decisions. In recent decades, the application of artificial intelligence (AI) and more specifically the branch of machine learning (ML) has led to a paradigm shift in the perceived utility of statistical modelling within healthcare. Within oesophageal cancer (OC) care, ML techniques have already been applied with early success to the analyses of histological samples and radiology imaging; however, it has not yet been applied to the MDT itself where such models are likely to benefit from incorporating information-rich, diverse datasets to increase predictive model accuracy.

**Methods:**

This review discusses the current role the MDT plays in modern UGI cancer care as well as the utilisation of ML techniques to date using histological and radiological data to predict treatment response, prognostication, nodal disease evaluation, and even resectability within OC.

**Results:**

The review finds that an emerging body of evidence is growing in support of ML tools within multiple domains relevant to decision-making within OC including automated histological analysis and radiomics. However, to date, no specific application has been directed to the MDT itself which routinely assimilates this information.

**Conclusions:**

The authors feel the UGI MDT offers an information-rich, diverse array of data from which ML offers the potential to standardise, automate, and produce more consistent, data-driven MDT decisions.

## Introduction

Oesophageal cancer (OC) is the 14^th^ most common cancer in the UK yet the 7^th^ commonest cause of cancer death.^1^ Only 39% of patients enter a curative pathway and less than 15% are alive at 5 years.^2,3^ Adenocarcinoma (OAC) of the oesophagus, in particular, has seen a 400% increase over the last 2 decades in part owing to the increased prevalence of gastro-oesophageal reflux and Barrett’s oesophagus and is now more prevalent than squamous cell carcinoma (OSCC) in some world regions including North America, Northern Europe, and Oceania.^4^

Gold standard management of OC remains curative resection, stage-permitting. Patients presenting with nodal disease also require neoadjuvant therapy (NAT) either as chemotherapy (NACT) or chemoradiotherapy (NACRT).^5^ Both have been shown to offer a survival advantage over surgery alone although to date, debate remains over which regime offers the better outcome.^5–9^ The Neo-AEGIS trial was intended to answer this very question, and yet 3-year follow-up data remains equivocal (despite a noticeably higher incidence of tumour regression grade (TRG) 1–2 within the CRT arm).^10^ Longer follow-up data is still awaited. The survival benefit from NAT, however, may not be conferred universally. A multicentre study of 1293 patients by Noble et al. demonstrated that a meaningful local response to NACT was only seen in those with TRG 1–2 (14.8% of the cohort) deemed “responders”. Overall survival in this group was 7.68 years versus 2.22 years in those with TRG 3–5 (85.2%).^11^ A major challenge is therefore predicting responders before starting NAT, although some groups have found modest success modelling variables available prior to surgery.^12,13^ Reliable predictive tools might then permit early triaging of non-responders directly to surgery in a bid to reduce NAT-associated morbidity and mortality for potentially little gain as it is recognised that NAT can decondition patients prior to surgery, potentially even rendering them inoperable.^14–16^

OC patients are consequently reliant on high-quality decision-making in often complex clinical contexts, with significant implications for their outcomes and quality of life.^17^ Currently, their treatment decisions are made by a multidisciplinary team (MDT), which is shown to improve patient outcomes.^18–20^ However, these services face ever-growing caseloads and clinical complexity, potentially leading to inconsistent and sometimes suboptimal decisions.^21^ Individual experience, perception, and bias can also lead to discordance within that decision-making consistency, effectively a form of “noise” in the process.^22^

Data-driven clinical decision tools are increasingly commonplace within medicine. The National Emergency Laparotomy Audit (NELA), for instance, has achieved widespread use for more objective operative risk stratification and the need for higher levels of care following emergency laparotomy.^23,24^ The domain of machine learning (ML) and by extension deep learning (a subset of ML which uses unstructured data, processing this through multiple “hidden layers” between the input and output layer to form a “neural network” designed to approximate human neural networks)^25^ offers huge potential to take ML a step further by standardising, optimising, and streamlining decision-making for cancer patients. Thus far, ML has been applied to decision-making with cardiac patients,^26^ breast cancer therapies,^27^ lung cancer,^28^ pancreatic cancer,^29^ and dermatological cancers.^30^ To date, no such approach has been made to the OC MDT. The purposes of this review are twofold: to contextualise the MDT’s role within OC and to discuss the applications of ML techniques within OC to date. This includes predicting treatment response using both histopathological and radiological data, as well as the emerging potential for radiomics for prognostication, nodal disease evaluation, and even resectability.

## Methods

Studies were selected on their use of, or discussion of artificial intelligence–based techniques on the UGI MDT as a whole or data types used by the MDT to determine treatment decisions for oesophageal cancer patients.

Studies will be further discussed by the modality of data they apply their machine learning approaches to. Within the MDT framework, the two main data sources outside of standard clinical patient information are histopathological and imaging based. This review will therefore discuss each of these separately.

Studies were obtained by a systematic search of PubMed using a combination of key terms including “Machine Learning”, “Artificial Intelligence”, “Oesophageal Cancer”, “Oesophagogastric Cancer”, “Esophageal”, “Esophagogastric”, “Upper Gastrointestinal Cancer”, “Upper Gastrointestinal Multidisciplinary team”, “Multidisciplinary team”, “Radiomics”, and “Predicting response”. Additional relevant studies were also identified through bibliographic examination of articles retrieved through the initial literature searches.

## The Multidisciplinary Team (MDT)

The clinical management of all cancer patients within the UK was centralised through MDTs following the Calman-Hine report in 1995.^31^ This brought together experts from all aspects of a patient’s care to focus on rapid, nuanced, complex, and above all-shared decision-making from the outset. MDTs comprise a variety of healthcare professionals: surgeons, physicians, oncologists, radiologists, histopathologists, specialist nurses, physiotherapists, occupational therapists, palliative care teams, and administrative staff. Centralisation also ensured adequate caseload to maintain clinical and operative skills. MDTs assess cancer site, stage, resectability, fitness for surgery, and necessary oncological adjuncts to formulate a treatment plan within the context of the patient’s wishes.

### Strengths of the MDT

**Fig. 1:**
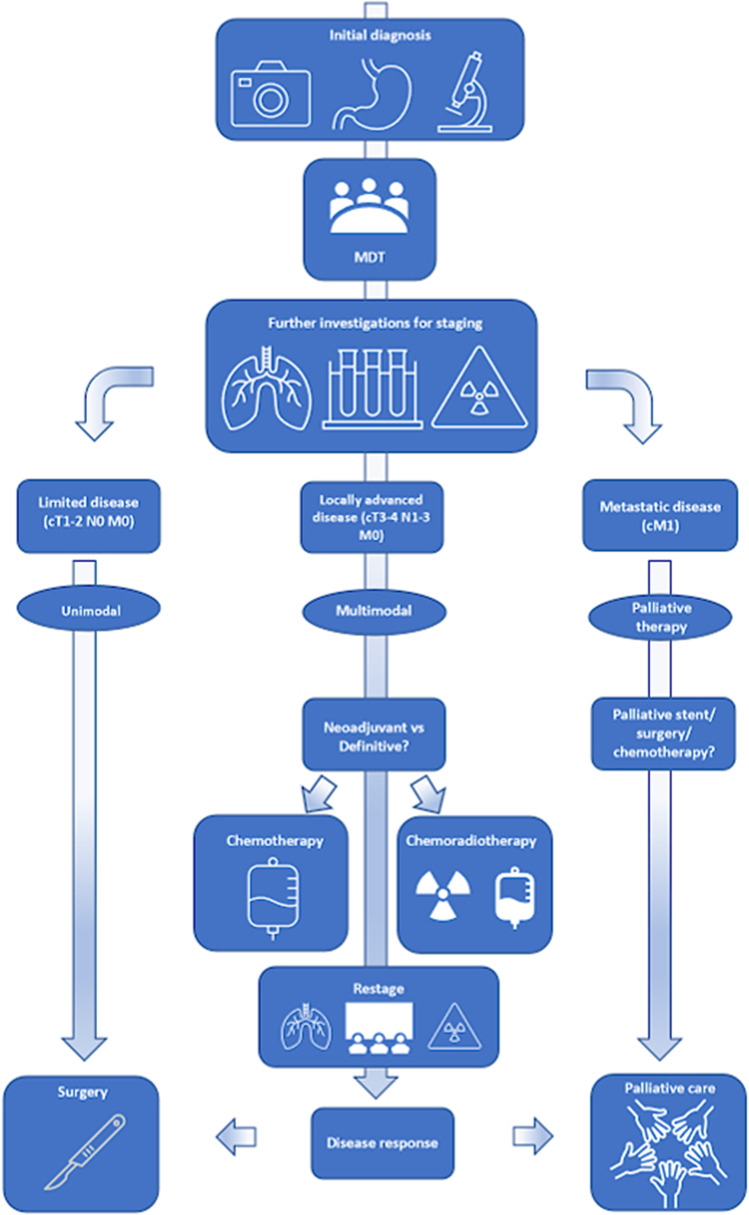
Schematic of the upper gastrointestinal (UGI) oesophageal cancer MDT decision-making process

Numerous studies have shown a benefit to managing oesophageal cancer via an MDT framework (Fig. 1) over surgeons managing such cases independently.^18–20,32^ They have been shown to reduce the incidence of open-and-close laparotomies or thoracotomies (from 21 and 5, respectively, to 13% and 0%, *p* = 0.02). Operative mortality is lower (5.7% vs 26%, *p* = 0.004), and 5-year survival is significantly higher (52% vs 10%, *p* = 0.0001). On multi-variate analysis, MDT management, lymph node metastases, and American Society of Anaesthesiologist (ASA) grade were all found to be independently associated with survival.^18^ Freeman et al. reported that a formal thoracic MDT for OC improved the rate of complete staging from 67 to 97% (*p* < 0.0001) and increased the percentage assessment by MDT from 72 to 98% (*p* < 0.0001) and adherence to national guidelines for management from 83 to 98% (*p* < 0.0001).^19^ Van Hagen and colleagues found that over one-third of management plans pre-conceived by individual clinicians as the “best course of action” for potentially curative upper gastrointestinal (UGI) cancer cases were still changed after MDT discussion^20^.

These benefits are not restricted to curative cases. A Dutch study of 948 palliative oesophagogastric (OG) patients found a significantly shorter time from diagnosis to commencement of palliative therapy in the MDT group (20 days vs 30 days, *p* < 0.001), a higher incidence of palliative external beam radiotherapy (EBRT) (38% vs 21%, OR 2.7), higher incidence of systemic therapy (30% vs 23%, OR 1.6,), fewer patients treated with palliative stents (4% vs 12%, OR 0.3), and greater duration of survival (169 days vs 107 days, HR 1.3).^32^ The authors attributed at least part of this improved survival to the greater usage of tumour-specific palliative therapies such as EBRT and systemic therapy in the MDT group.

### Vulnerabilities of the MDT

Despite the multitude of strengths of the MDT system, it is also vulnerable to clinical, inter-personal, and logistic challenges. Rising caseloads, reduced dedicated MDT time, missing data, patient complexity, and inter-member disagreement all lead to inconsistent and suboptimal decision-making with potentially life-limiting consequences for a patient’s health and quality of life.^33^ The dedicated preparation time required and associated financial cost are also considerable. Each hour of an MDT has been estimated to take 2 h for a radiologist and 2.4 h for a histopathologist to prepare for.^34^ A systematic review in 2011 exploring clinical, social, and technological factors influencing MDT decision-making found that definitive plans were only reached at first discussion in 47.6–73% of cases owing to time pressures or inadequacy of available information at the time of discussion (e.g., imaging, staging, pathology review, or patient comorbidities).^21^ A failure to implement MDT decisions was seen in 1–16% of cases owing to differing patient wishes or inappropriate management plans when factoring in patient comorbidities. General surgical, urological, and soft tissue cancer MDTs were found to have clinician-made decisions based almost entirely on clinical information. The review noted that physicians drove the decision-making, often ignoring nurse-led input usually at the detriment of the overall efficacy of the MDT.

Patient-centred decision-making varies within MDTs. Another study by Lamb et al. determined that patient wishes were infrequently considered at MDT unless nurses present could, and felt empowered to, speak up.^35^ Furthermore, essential social data such as a patient’s social position, attitude, values, and preferences often be missing, incomplete, or selectively presented in order to influence the discussion in a particular direction.^36^

Leadership and personal biases are salient factors. A study of breast cancer MDTs found that while a lack of clarity and conflict over leadership were negative predictors for effective internal communication, team effectiveness, and resource efficiency, a single strong leader also harmed innovation.^37^ Their results further highlighted that perceptions of team effectiveness could vary significantly by role, noting that breast surgeons and breast care nurses consistently rated their MDT’s performance higher than their radiology and histopathology counterparts.

Such vulnerabilities can have clinical impact on OC patients. A small observational multicentre Danish study investigated inter-observer variability between MDT decisions at four major UGI cancer units in Denmark.^22^ The study presented 20 OSCC cases as new referrals to each of the four centres to determine resectability, curability, and treatment strategy. The authors reviewed the frequency by which disagreement between MDTs resulted in a different treatment recommendation and whether this had a clinical impact. Moderate concordance was seen on classifying T-stage, M-stage, resectability, and curability, while N-stage and operability only reached “fair” concordance. The authors traced much of the disagreement back to classifying “Mx” and consideration of “further investigations”. The biggest impact of their findings was however that MDT disagreement led to a clinical impact in 60% of cases. The authors reported that while operability was crucial to determining an accurate treatment strategy, it was most vulnerable to inter-observer differences. Yet given the clinical information available at MDT, it remained difficult if not almost impossible to determine accurately at the time of the meeting. The authors reinforced the importance of being able to establish operability either pre-MDT or with additional data variables available at the time of discussion.

Similarly, comorbidity is inadequately presented or integrated into cancer MDTs, despite having a substantial impact on the quality of its judgements. A 2015 systematic review found that comorbidities were; not well considered (meaning MDTs were less likely to reach a treatment decision); were often the reason given for deviating from treatment guidelines; and where a treatment recommendation was given, was usually the reason it was not implemented.^33^

### Decision-Making Within OC

Table 1 outlines the 2018 National Institute for Clinical Excellence (NICE) guidelines for the management of OC.^38^ Notably while some authors categorise T2N0 disease as early and amenable to endomucosal resection (EMR), NICE supports the use of NAT in this cohort, likely to minimise local recurrence risk from micro-metastases.^39,40^ It can be readily appreciated that histology, TNM staging, and an assessment of patient fitness (commonly quantified by the WHO Performance Status classification) account for the bulk of decision critical parameters. While the concept of comorbidity is acknowledged, especially when determining suitability for palliative chemotherapy, such guidelines remain simplistic, rarely factoring in dimensions such as high-risk comorbidities, social variables, or even ease of patient access to CRT centres.

**Table 1:** 2018 NICE guidelines for the management of OC ^38^

Disease stage	OAC	OSCC
T1aN0	Offer EMR	Offer EMR
T1bN0	Offer surgery	Offer either - Definitive CRT - Surgical resection
T2-4 N0-3 M0	Offer either: - NACT ± ACT - NACRT Assess response Then surgery	Offer either -Radical CRT Or: - NACRT Assess response Then surgery
Non-metastatic disease unsuitable for surgery	Consider - CRT if feasible within RT field Or: - Chemotherapy - Stenting - Palliative RT - Best supportive care	Consider - CRT if feasible within RT field Or: - Chemotherapy - Stenting - Palliative RT - Best supportive care
Metastatic disease	If HER2 + ve: - Trastuzumab (Herceptin) 1^st^ line palliative chemotherapy (if performance status 0–2, no significant comorbidities) 2^nd^ line palliative chemotherapy	If HER2 + ve: - Trastuzumab (Herceptin) 1^st^ line palliative chemotherapy (if performance status 0–2, no significant comorbidities) 2^nd^ line palliative chemotherapy

*OAC*, oesophageal adenocarcinoma; *OSCC*, oesophageal squamous cell carcinoma; *EMR*, endomucosal resection; *CRT*, chemoradiotherapy; *NACRT*, neoadjuvant chemoradiotherapy; *NACT*, neoadjuvant chemotherapy; *ACT*, adjuvant chemotherapy; *HER2*, human epidermal growth factor 2

## A Role for Machine Learning?

Machine learning (ML) has gained popularity within healthcare environments for its potential to assist clinical decision-making by detecting complex patterns within large datasets. Great promise has been shown even in OC, in predicting outcomes following oesophagectomy.^41^ However, while post-operative models have shown good discrimination and calibration, pre-operative models are more challenging.^12^ Despite this, the pre-treatment MDT discussion remains a key mile marker in the patient’s care pathway, and optimising the decision-making at this stage is vital. MDTs typically assimilate information from clinical, pathological, and radiological sources, each of which offers a potential focus for ML applications, yet surprisingly, this has not been exploited in UGI MDTs to date.

Machine learning is traditionally divided into supervised and unsupervised learning. Supervised learning requires the “labelling” of data (the ground truth is given to the machine). The machine is then able to compare the input and outcome data to determine the best fitting model which explains any underlying structure of the data. Supervised learning is thus well suited to smaller datasets where the ground truth is known—a prime example being the outcomes of historic MDTs where treatment decisions of patients are already known. By comparison, unsupervised learning algorithms identify patterns within datasets to extract features that may speak to their structure. Such techniques are useful when the ground truth is unknown, necessitating large volumes of data—a challenge frequently encountered in cancer datasets. Models are trained using data partitioned from the main dataset, by which the machine searches for patterns between the selected variables and the designated outcome. Ideal models learn from training data to make accurate predictions when fed new unseen data (testing datasets), minimising “under-” or “over-fitting”. Under-fitted models are too simplistic or inflexible to capture the underlying relationships leading to high error rates in both training and testing (bias). Over-fitting occurs when the model features are too numerous or complex resulting in high variance. These models perform well within training but struggle on test/validation sets.^42^ This may be mitigated by increasing the size of the training set available and the diversity of the observations themselves, making it more representative of the theoretical population distribution. In real-world settings however, this is often difficult with health data especially for rarer clinical scenarios under study. Table 2 summarises some common ML-based techniques.

**Table 2:** Common machine learning techniques

	Summary	Benefits	Drawback
Decision trees	Flowchart-based modelling whereby variables are trialled at each “node” of a tree (decision split point) to determine the best combination of root, branch, and leaf nodes for the overall model	Provides an interpretable model and easy to visualise No assumptions made about data distribution Can manage regression and classification tasks	Less well suited to continuous variable outcomes Produces a single tree but may be computationally expensive to grow tree as must trial every split of variables at each node Prone to over-fitting, especially if large number of variables and small datasets
Random forest	A tree-based modelling technique which aggregates hundreds of individual decision trees, each composed of a random selection of predictor variables	Copes with large feature pools Randomly selecting a subset of variables for each tree rather than the full pool minimises over-fitting and increases generalisability Can be used to assist feature selection based on relative importance of each variable	Sacrifices interpretability for overall model performance Vulnerable to outliers within dataset
Ridge regularisation	Also known as L2 regularisation, a form of regularisation method which acts to minimise a loss function (a penalty associated with misclassification)	Ridge regularisation produces a more generalisable regression model by shrinking variable coefficients to reduce model over-fitting	Ridge regression never shrinks coefficients to “0”, thus maintaining all variables within the model. This in turn reduces interpretability
Least absolute shrinkage and selection operator (LASSO)	Also known as L1 regularisation. Similar approach to ridge regression, however the penalty function is derived from the absolute sum of the coefficient as opposed to their square as is used in ridge	Allows automatic feature selection LASSO allows coefficients to be shrunk to “0” and effectively drops them from the model which allows for feature elimination Used to minimise model over-fitting	In situations where predictors outnumber the observations, LASSO will reduce variable pools even if non-significant variables are nevertheless relevant to the model as a whole Similarly, where variables may be correlated, LASSO may randomly select one and eliminate the other
Logistic regression	Form of regression analysis for outcomes which are categorical (and often binary). Learns a linear relationship in form *y* = *c* + *β*_1_*x* + *β*_1_x + *β*_1_x + … *β*_*n*_*x* to predict probability of a given class	Provides an interpretable model The variable coefficient enumerate the relative weights of each variable to the overall model and direction Easy to train and computationally inexpensive	Requires linearity between the predictors and outcomes Observations need to be independent of each other Limited to categorical outcome prediction
Support vector machine	Segregates data by creating a decision boundary of “hyperplane” to allow class separation	Useful in binary outcome predictions Capable of handling high-order data relationships Commonly used in radiomic tasks	For more complex higher-order data, requires elevation of data into higher dimensions to achieve hyperplane
Convolutional neural network	Uses multiple “hidden layers” of processing to analyse input data and provide a task outcome. Deep learning models are formed around the concept of recreating neural networks—comes under ML discipline of deep learning	Powerful ML approaches Particularly suited to complex tasks such as audio and image analysis	Computationally intense Requires large volume datasets Sacrifices interpretability for overall model performance

## ML Applications Within OC to Date

### Histopathological Analysis

The application of ML to histopathology in order to augment decision-making in clinical care is gaining popularity.^43–45^ RNA and whole genome sequencing (WGS) offer detailed and individualised data for analysis at the cost of expensive tissue analytical processes.^41^ Computer vision–based ML promises comparatively low-cost, automated large-scale analysis in OC, although to date very few studies have applied such techniques to OC (Table 3).^41,46^ Pilot work using convolutional neural networks (CNN) to process unlabelled high-resolution digital OAC histology slides achieved good internal validation in predicting response to NAT (C-index 0.836).^41^ While these results are promising, validation over larger datasets and external data sources remains necessary before use in clinical practice, especially as the use of unsupervised learning creates a “black box” solution impeding transparency, “explainability”, and ultimately trust in the solution. An additional confounder in the Rahman et al. study was the use of both NACRT and NACT within the patient cohort. The training of the CNN in this instance utilised ImageNet (non-specific images from a vast online database of everyday images) in the form of transfer learning. This circumvented the need for the sheer volume of histology-specific training images otherwise needed to produce a sufficiently accurate model. Pre-trained networks have performed competitively against models trained from scratch.^47^ However, with academic collaborative projects such as the Northern Pathology Imaging Co-operative looking to accumulate large-scale digital pathology repositories, this challenge may become more achievable in the future, especially as transfer learning is unlikely to be sufficiently robust for routine clinical use.

**Table 3:** Studies applying ML to histopathological data within OC

Study	Country	Study size (*n*)	Histology	Image modality	ML techniques	Outcome predicted	Model performance metric	Results
Rahman et al. (2021) ^41^	UK	46	Mixed	WSI with patch conversion	CNN (Xception) + elastic net regression	Response to neoadjuvant therapy (NACT/NACRT) comparing histopathological analysis vs RNAseq	AUC	AUC for histopathology slide features 0.763 vs RNAseq (0.782) AUC for segment slides exceeded both (0.870)
Tomita et al. (2019) ^46^	USA	180	AC, BE, and dysplasia	WSI with patch conversion	CNN (ResNet-18) + attention-based neural network	Classification of Barrett’s ± dysplasia and oesophageal adenocarcinoma comparing tissue-level annotations vs traditional ROI segmentation	Accuracy, recall, precision, and *F*1 score	Mean accuracy of 0.73 for differentiating BE, BE + dysplasia, and AC *F*1 scores for differentiating BE, BE + dysplasia, and AC were 0.72, 0.30, and 0.67, respectively

*AC*, adenocarcinoma; *BE*, Barrett’s oesophagus; *WSI*, whole slide image; *AUC*, area under receiver operator characteristic curve; *NACRT*, neoadjuvant chemoradiotherapy; *NACT*, neoadjuvant chemotherapy; *ANN*, artificial neural network; *CNN*, convolutional neural network; *RNAseq*, sequenced ribonucleic acid

With only a minority of OC patients benefitting from NAT, it is appealing for MDTs to be able to identify them as early as possible. Accurate prediction of tumour response from initial biopsies usually available at the beginning of a referral pathway would allow patients to be filtered towards the most beneficial therapy in the timeliest fashion.

### Imaging-Based Approaches—Radiomics

Over the last two decades, advances in image processing and analysis have allowed the field of radiomics to flourish developing a substantial evidence base across numerous solid organ cancer types.^48^ Radiomics refers to the extraction of quantitative, clinically significant, high-dimensional imaging biomarkers from standard-of-care medical imaging to predict a range of clinical outcomes.^42^ Standard radiological assessments within MDTs for OC are traditionally largely qualitative, with some quantification of tumour size, number, and position of suspected lymphadenopathy and the presence of distant metastases. A human eye–based assessment however may struggle to pick out additional hidden data on a pixel/voxel level within the image stacks and inherently involves a degree of both selection bias as well as inter- and intra-observer variability.^49^ Radiomics seeks to mine this data for more tailored decision-making. Coupling this to the MDT infrastructure would benefit OC patients by achieving highly detailed assessment of their disease burden, resectability, and probable interval response to NAT at a very early stage.

#### Radiomic Workflow

**Fig. 2:**
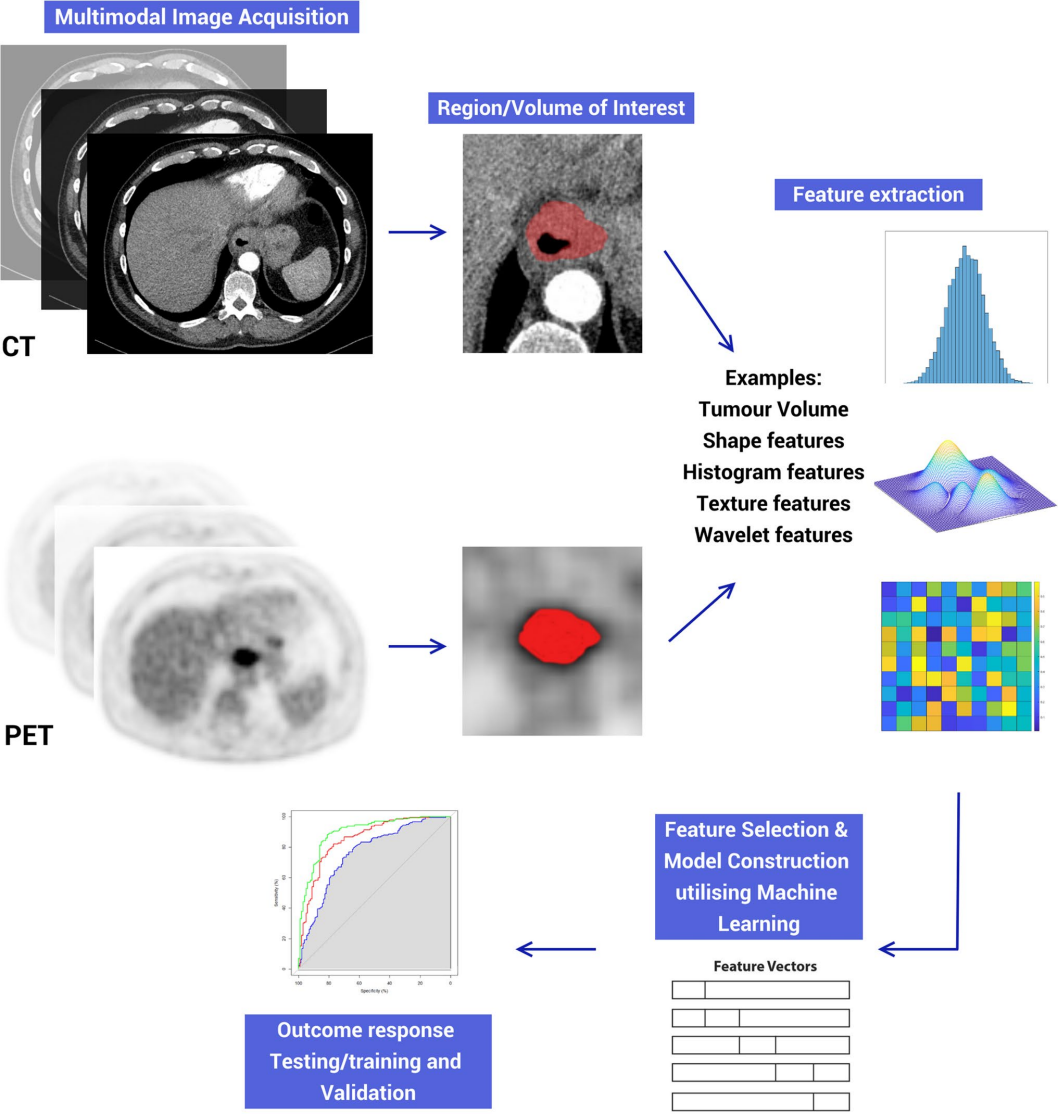
A standard radiomic workflow

The radiomic workflow (Fig. 2) can be summarised as image acquisition, image pre-processing, segmentation, feature extraction, data preparation, feature reduction, and model development.^42,50^ Image acquisition relates to the curating of imaging stacks containing regions of interest (ROI) under investigation. Features extracted from ROIs may mirror the tumour phenotype and its molecular fingerprint.^49^ Image pre-processing includes segmentation of ROIs which may be manual (considered gold standard but resource intensive), automatic, or hybridised. Automated segmentations while potentially error-prone offer workflow automation with reasonable accuracy.^51^ The next step is feature extraction which is the functional core of radiomics. Visual features embedded within images are extracted and converted into quantifiable vectors.^42,49^ Vectors may differ in scales; thus, data preparation includes feature scaling, data continuation, discretisation, and under- or over-sampling for class imbalances.^52^ The resultant features may be hundreds in number and counter-productive to a well-performing model.^53^ Dimensionality reduction and feature selection can minimise those redundant, non-relevant features which may slow a model for little gain.^54–56^ The final feature pool which forms the radiomic model is then used to classify groups of patients into one of several outcome classes, whether this is based on a perceived risk or intervention outcome. Finally, validation of the generated model must then be done internally and externally as it speaks to the generalisability of the final model.^57^

#### Radiomics in OC

An evolving body of evidence is now emerging for OC in predicting treatment response, prognosis, nodal status, and even resectability.^16^ Improving the diagnostic accuracy of each of these aspects in turn using radiomics can drive forward a large portion of the MDT’s weekly workflow. Table 4 summarises studies which have applied radiomics to the OC domain.

**Table 4:** Studies applying radiomic techniques within OC

Study	Country	Study size (*n*)	Histology	Imaging modality	ML techniques	Outcome predicted	Model performance metric	Results
Ou et al. (2019) ^58^	China	591	SCC	CT	LASSO, logistic regression, RF, SVM, XGBoost, and decision trees	Resectability of SCC	AUC, accuracy, and *F*1 score	Logistic regression radiomic model performed best (validation set AUC 0.87 ± 0.02, accuracy 0.86, F1 score 0.86)
Hou et al. (2017) ^59^	China	49	Mixed	CT	SVM and ANN	Therapeutic response to NACRT	AUROC	Radiomics based SVM AUC 0.891 and ANN AUC 0.972 for responders vs non responders Skewness and Kurtosis features capable of differentiating partial response and stable disease and Kurtosis also discriminatory for partial versus complete response
Larue et al. (2018) ^60^	Netherlands	239	Mixed	CT	RF	3-year survival post NACRT	AUROC (95% CI)	Radiomics RF model validation set AUC 0.61 (0.47–0.75) vs clinical parameter RF validation set AUC 0.62 (0.49–0.76)
Tan et al. (2019) ^61^	China	230	SCC	CT	LASSO and logistic regression	Predicting LN metastases in resectable SCC	Discrimination, calibration, and reclassification	AUC of model combining radiomic signature with CT LN status was 0.773 Discrimination of signature significantly better vs LN size criteria alone (*p* = 0.005)
Beukinga et al. (2017) ^62^	Netherlands	97	Mixed	PET/CT	LASSO and logistic regression	pCR following NACRT	AUC	Model combining clinical parameters with PET/CT derived textural features outperformed SUVmax models (AUC 0.74 vs 0.54 on internal validation)
Simoni et al. (2020) ^63^	Italy	54	Mixed	PET/CT	Logistic regression	Pathological response to NACRT	ROC	MTV (AUC 0.74) and TLG (AUC 0.69) correlated with tumour regression at baseline PET SUVmean (AUC 0.67) and TLG (AUC 0.64) related to higher chance of significant pathological response at second PET after induction chemotherapy
Cao et al. (2020) ^65^	China	159	SCC	PET	LASSO and logistic regression	Treatment response following CCRT	AUC	Validation set AUC for radiomic signature–based model was 0.835
Zhang et al. (2014) ^66^	USA and China	20	Mixed	PET/CT	SVM and logistic regression	Pathological tumour response to NACRT	AUC (95% CI)	SVM combining classic PET/CT measures + clinical parameters + spatiotemporal PET features reached AUC of 1.0 vs 0.56 (0.07), 0.6 (0.06), and 0.94 (0.02) individually SVM additionally outperformed LR (combined model AUC 0.9 (0.06))
Qiu et al. (2020) ^67^	China	206	SCC	CT	LASSO and Cox proportional hazards	Recurrence free survival following pCR post NACRT	Validation set C-index (95% CI)	AUC 0.724 (0.696–0.752) with radiomics + clinical risk factors model vs radiomics (0.671, 0.624–0.718) or clinical risk factors (0.629, 0.597–0.661)
Yang et al. (2019) ^68^	Taiwan	548	SCC	PET	18/34-layer CNN	1-year survival post-diagnosis	AUC	AUC of 0.738 Patients predicted to expire at 1 year who survived had a lower 5-year survival than those predicted to survive 1 year (32.6% vs 50.5%, *p* < 0.001)—authors inferred that the CNN model also reflected aggressive tumour biology

*MTV*, metabolic tumour volume; *TLG*, total lesion glycolysis; *SUV*, standardised uptake value; *PET*, positron emission tomography; *CT*, computerised tomography; *AUC*, area under receiver operator characteristic curve; *LASSO*, least absolute shrinkage and selection operator; *SVM*, support vector machine; *pCR*, pathological complete response; *NACRT*, neoadjuvant chemoradiotherapy; *NACT*, neoadjuvant chemotherapy; *CCRT*, concurrent chemoradiotherapy; *RF*, random forests; *XGBoost*, extreme gradient boosting; *ANN*, artificial neural network; *CNN*, convolutional neural network

##### Treatment Response Evaluation

Most studies predicting treatment response have focussed on NACRT rather than NACT, using OSCC primarily or mixed histology datasets.^58–61^ As many of these studies originate from China, where 90% of OC is the OSCC subtype, this is unsurprising. Nonetheless, it has long been appreciated that tumour heterogeneity on imaging is associated with aggressive tumour biology and impaired treatment response in OC leading to many ML techniques being applied to this very issue.^62^ As imaging is often one of the earliest potential sources of information on tumour biology for OC patients, accurate characterisation here can tailor the oncological plan even before histology has been returned.

Flurodeoxyglucose (^18^F)-positron emission tomography (FDG-PET) is used to assess for metastatic disease by uptake of FDG in metabolically active cells. Metabolic tumour volume (MTV) and standardised uptake value (SUV) on FDG-PET may variably predict response to NACRT in OC across serial imaging time points as well holding prognostic significance for survival.^16,63,64^ One PET study drew inspiration from DNA microarray analysis combining an extracted radiomic signature with a LASSO-logistic regression model to predict treatment response (AUC 0.835). While the authors contended with a class imbalance favouring responders and a radiomic signature derived from only 20 patients, the approach was nevertheless an intriguing one.^65^ A drawback to FDG-PET is its expense, time consumption, and lack of the complete molecular characterisation that one wishes to exploit when mining spatial heterogeneity in tissue architecture and metabolic activity.^62^ Contrast-enhanced CT is comparatively ubiquitous in day-to-day clinical practice for assessing treatment response; it is quick and easily accessible. In smaller case series, it has even successfully predicted response to NACRT using as few as five shape and histogram-based metrics (AUC 0.686–0.727).^59^

Studies combining multimodal data frequently show superior performance compared to single data streams alone. Zhang et al. predicted pathological tumour response to NACRT in OC patients applying both logistic regression (LR) and support vector machine (SVM) models finding that a combination of conventional PET/CT response measures, clinical data (TNM, histology, patient demographics), and spatial–temporal PET/CT features offered superior predictive performance over individual feature sets (AUC of 1.0 for SVM vs 0.9 for LR).^66^ However, the study did not factor in nodal disease and was small (*N* = 20), thus risking over-fitting in the absence of external validation. Another study combining clinical information, geometry, PET textural features, and CT textural features used a LASSO-regularised LR model to produce an AUC of 0.78 versus 0.58 for SUVmax alone.^62^

##### Prognostication

A number of studies have attempted to prognosticate in OC. Qiu et al., for instance, reported disease recurrence in one-third of patients who experienced a pathological complete response following NACRT and surgery for OSCC.^67^ Their CT-based nomogram combined clinical risk factors and a radiomic signature of eight features. This proved superior (C-index of 0.746) versus radiomic (0.685) and clinical (0.614) features alone (*p* < 0.001 in all cases). The model could effectively stratify patients into high and low risk categories potentially offering tailored adjuvant therapy post-resection.

One Dutch study predicted 3-year survival after NACRT using a random forest model comparing clinical and radiomic feature sets on pre-treatment CT. This study did include both OAC and OSCC, albeit heavily weighted towards the former.^60^ They reported an AUC of 0.61 on external validation for their radiomic model versus 0.62 for their clinical dataset. While the authors did show clear survival differences between TRG 1–2 and TRG 3–5 patients within the study cohort, this did not translate to a statistically significant difference in survival within validation sets when risk was stratified by the model again reflecting the Neo-AEGIS trial.^5^

Deep convolutional neural networks (CNN) have also proved capable of predicting 1-year survival in OSCC when trained on PET images. A Taiwanese study pre-trained a ResNet 3D CNN using a mixed set of 1,107 OSSC and lung cancer PET scans.^68^ Their best model attained an AUC of 0.738, outperforming clinical data alone. The authors found that CNN predictions themselves were significant on multivariable analysis for survival indicating that meaningful prognostic hidden data could be extricated. The authors did recognise that the extraction and selection of features was not transparent, i.e. a “black box” problem.

While accurate knowledge of operability and treatment response is vital for counselling patients of MDT treatment recommendations, precise prognostication allows them to contextualise the cost–benefit balance. The studies described above therefore highlight the significant role ML can play here.

##### Nodal Status

The prediction of lymph node (LN) disease conveys implications for prognosis and MDT treatment decisions. Tan and colleagues achieved a test set validation AUC of 0.773 using LASSO-LR when predicting LN metastases in resectable OSCC cases, outperforming size criteria alone on CT imaging.^61^ Another CT-based study reported near-identical performance in testing using an elastic net approach across what was implied to be a mixed histological cohort.^69^

##### Other Outcomes

Less conventional radiomic–based problems have also been explored. Resectability, for example, was predicted in one study of 591 OSCC patients. A LASSO-enhanced dimensionality reduction technique across multiple ML algorithms showed that multivariable logistic regression (MLR) offered the best performance (AUC 0.87, accuracy 0.86).^58^ Another study in radio-genomics used CT imaging to help predict microRNA-1246 expression, a biomarker linked with prognostic significance in OSCC.^70^ Correlation analysis extracted image features correlating with miR-1246 levels in 92 patients. Linear regression then separated patients into low and high expression correlating with survival. Unfortunately, while miR-1246 levels were significantly raised in stage 2 disease, no difference was seen between healthy controls and stage 1 disease, thereby limiting miR-1246’s potential for screening.

## Challenges and Future Directions for ML and the MDT

One of the main challenges facing ML tools designed for the MDT is inevitably the degree of noise within the datasets. This may be attributable to several factors such as variation in attendance of specific MDT members, the allocated time they possess to be present and discuss each case, clinical equipoise over treatment options, clinician preferences, and even social factors such as patient geography and their relationships to high-resource units.^35^ Incorporating some or all such factors into future model training may adjust for this noise. Trustworthiness and transparency remain another key issues for model deployment within healthcare settings. Patients, clinicians, and health regulators alike will likely require a degree of explainability for ML solutions. A route through this would be to focus on more simplistic and/or explainable models such as logistic regression and decision tree algorithms (a process which falls under explainable AI or “XAI” ^71^). However while XAI intuitively fits the perception of providing understanding of a system’s decisions, inherently explainable algorithms and post-hoc explainability tools may conversely reflect a misleading sense of true trustworthiness, with patient safety potentially better achieved through robust validation techniques instead.^72^ Once model performance is confirmed at a single unit, the tool may then be extended to other MDTs. This may be through tailoring a model to each unit individually or applying a single model to multiple units. The former approach is labour intensive yet minimises under-fitting or poor generalisability as we are no longer modelling noise and idiosyncrasies particular to one MDT and applying these “rules” to another. Alternatively, a one-size-fits-all model may be designed for generalisation across multiple provided the practices of each such unit follow a consistent pattern. To achieve this, the training data requires amalgamation and homogenisation from multiple sources which pose challenges such as data sharing agreements between centres, standardised patient data acquisition, and navigating the variation in imaging protocols associated with each individual hospital.^50^ Daramola et al. propose a multimodal AI framework for amalgamation, processing, and model development using similar data types in managing infectious diseases within sub-Saharan Africa.^73^ Through these approaches, ML allows OC MDTs to automate aspects of their workflow, potentially extract clinically meaningful information from imaging data, and streamline decision-making which has been learned from its historic decision-making framework. As UGI MDTs also manage gastric cancers, the concept is also transferrable to their gastric cancer patients and potentially other solid body cancers.

## Conclusion

The OC MDT handles complex treatment decisions with potentially life-altering implications for its patients, increasingly under pressures of modern practice and caseloads. ML has shown great promise as an assistive tool in many clinical domains. While ML approaches have been applied to several data types relevant to the OC MDT, the MDT itself is as yet an unexplored arena. Future work should now look to integrate these techniques to streamline and assist the MDT’s own decision-making. This in turn may offer the capacity to offer data-driven solutions, reduce costs and help prioritise their caseload, and thereby positively impact patient cancer care.

## Data Availability

Data availability was not applicable to this article.
